# Analysis of *ADORA2A*, *MTA1*, *PTGDS*, *PTGS2*, *NSF*, and *HNMT* Gene Expression Levels in Peripheral Blood of Patients with Early Stages of Parkinson's Disease

**DOI:** 10.1155/2023/9412776

**Published:** 2023-11-20

**Authors:** Ekaterina I. Semenova, Suzanna A. Partevian, Marina V. Shulskaya, Margarita M. Rudenok, Maria V. Lukashevich, Nina M. Baranova, Olga B. Doronina, Kseniya S. Doronina, Anna V. Rosinskaya, Ekaterina Y. Fedotova, Sergey N. Illarioshkin, Petr A. Slominsky, Maria I. Shadrina, Anelya Kh. Alieva

**Affiliations:** ^1^National Research Centre “Kurchatov Institute”, 2 Kurchatova Sq., 123182 Moscow, Russia; ^2^Peoples' Friendship University of Russia (RUDN University), 6, Miklukho-Maklaya Str., 117198 Moscow, Russia; ^3^Novosibirsk State Medical University, 52, Krasnyy Ave., 630091 Novosibirsk, Russia; ^4^State Public Health Institution Primorsk Regional Clinical Hospital No. 1, 57 Aleutskaya St., 690091 Vladivostok, Russia; ^5^Research Centre of Neurology, 80, Volokolamskoye Shosse, 125367 Moscow, Russia

## Abstract

Parkinson's disease (PD) is a common chronic, age-related neurodegenerative disease. This disease is characterized by a long prodromal period. In this context, it is important to search for the genes and mechanisms that are involved in the development of the pathological process in the earliest stages of the disease. Published data suggest that blood cells, particularly lymphocytes, may be a model for studying the processes that occur in the brain in PD. Thus, in the present work, we performed an analysis of changes in the expression of the genes *ADORA2A*, *MTA1*, *PTGDS*, *PTGS2*, *NSF*, and *HNMT* in the peripheral blood of patients with early stages of PD (stages 1 and 2 of the Hoehn–Yahr scale). We found significant and PD-specific expression changes of four genes, i.e., *MTA1*, *PTGS2*, *NSF*, and *HNMT*, in the peripheral blood of patients with early stages of PD. These genes may be associated with PD pathogenesis in the early clinical stages and can be considered as potential candidate genes for this disease. Altered expression of the *ADORA2A* gene in treated PD patients may indicate that this gene is involved in processes affected by antiparkinsonian therapy.

## 1. Introduction

Parkinson's disease (PD) is a common chronic, age-related neurodegenerative disease [1, 2]. According to the data for 2015, there were more than 6 million PD patients globally [3]. Moreover, the number of individuals with PD is expected to double by 2030 [4].

The development of PD is primarily associated with the loss of dopaminergic (DAergic) neurons of the *substantia nigra* (SN) *pars compacta*, resulting in the classic motor symptoms [5]. These symptoms are tremor, rigidity, bradykinesia, and postural instability [6]. It should be noted that the manifestation of motor symptoms occurs only after the loss of about 70% of DAergic neurons, and it can begin more than 20 years from the onset of the neurodegenerative process [7, 8]. A long prodromal period is the main reason for late diagnosis of PD and, accordingly, for late initiation of therapy. In this regard, it is important to search for the genes and mechanisms that are involved in the development of the pathological process in the earliest stages of the disease. This task is also urgent because there is still no easy-to-follow laboratory diagnostic method based on biochemical parameters that could predict the risk of PD with high accuracy [9]. Early detection of PD is essential for initiating timely treatment and slowing the progression of the disease.

It is known that the main pathological processes in PD occur mainly in the central nervous system. Published data suggest that blood cells, particularly lymphocytes, may be a model for studying the processes that occur in the brain in PD. It has been shown that genes associated with DAergic signal transduction are expressed in blood cells, as well as in DAergic neurons [10–14]. In addition, some of the gene products expressed in peripheral blood may reflect molecular events associated with PD pathogenesis. Changes in mRNA and protein levels associated with ubiquitin-proteasome protein degradation [15–18], mitochondrial dysfunction [16–19], oxidative stress [18, 20], apoptosis [16, 17, 21, 22], and autophagy [23] have been described in peripheral blood cells from PD patients. Thus, analysis of individual genes expression changes at the mRNA level in peripheral blood may be important in the study of early stages of PD.

Our previous studies have resulted in the identification of potential candidate PD genes. Previously, we found a significant change in the expression of three genes associated with circadian rhythms, *ADORA2A*, *MTA1*, and *PTGDS*, in the peripheral blood of twins with PD [24], as well as *PTGS2*, associated with inflammatory processes, in fibroblast samples from twins with PD [25]. When studying mice with an MPTP-induced model of the early stage of PD, we obtained interesting results on changes in the expression of the *NSF* gene, the protein product of which is involved in transport processes [26]. In addition, the *HNMT* gene was selected by analyzing the published data. This gene encodes an enzyme of histamine metabolism, and it is noteworthy for analysis because it has been repeatedly reported that changes in the histaminergic system are observed in PD [27, 28]. Thus, in the present work, we focused on studying changes in the expression of *ADORA2A*, *MTA1*, *PTGDS*, *PTGS2*, *NSF*, and *HNMT* genes in the peripheral blood of patients with early stages of PD.

## 2. Materials and Methods

### 2.1. Patients

In the present work, 56 patients with early stages of PD (stages 1 and 2 of the Hoehn–Yahr scale; 25 men and 31 women; mean age ± standard deviation 58.6 ± 10.8 years) were studied. There were 47 untreated and 9 treated patients. This sample included patients who were examined at Novosibirsk State Medical University and State Public Health Institution Primorsk Regional Clinical Hospital No. 1 in Vladivostok. For clinical assessment of PD, patients were studied using the International Uniform Assessment Scale for PD (Unified Parkinson's Disease Rating Scale, UPDRS) [29] and Hoehn–Yahr scale [30]. Only those patients who lacked the most frequent PD-associated mutations were selected for analysis. Patients with PD who were treated received different medications with dopamine receptor agonists (Requip, Pronoran) or L-dopa (Madopar, Stalevo) either as monotherapy or in various combinations. The comparison group included 44 neurologically healthy volunteers (mean age ± standard deviation 50.0 ± 12.4 years). In addition, 23 patients with various neurological diseases were selected as an additional control to the “neurological control” group (mean age ± standard deviation 46.1 ± 14.5 years). A detailed description of the comparison groups is presented in our earlier work [31]. The studied patients and healthy volunteers were of Slavic origin. All blood samples were collected with the informed consent of the investigated subjects. The study was conducted in accordance with the World Medical Assembly (WMA) Declaration of Helsinki-Ethical Principles for Medical Research Involving Human Subjects [32]. The study was approved by the Ethics Committee of the Research Center of Neurology, Institute of Molecular Genetics of National Research Centre “Kurchatov Institute,” and Novosibirsk State Medical University.

### 2.2. Total RNA Isolation from Peripheral Blood and Expression Analysis of Individual Candidate Genes

Total RNA isolation from peripheral blood was performed, according to the previously described protocol [33]. After isolation, yeast tRNA at a concentration of 1 mg/mL was added to the resulting total RNA solution for protection [34]. Analysis of mRNA levels using reverse transcription and real-time PCR (TaqMan technology) was carried out in accordance with the protocols described previously [35].

### 2.3. Statistical and Bioinformatic Data Processing

Primer and probe sequence design was produced using Beacon designer 7.0 software (Premier Biosoft International, Palo Alto, CA, United States) and nucleotide sequences of the candidate genes *MTA1*, *ADORA2A*, *PTGDS*, *AHCY*, *HNMT*, *NSF*, and *PTGS2* and housekeeping genes *SARS1* and *PSMD6* from the NCBI database [35]. The sequences of gene-specific primers and probes for the *MTA1*, *ADORA2A*, *PTGDS*, *SARS1*, and *PSMD6* genes are listed in our previous work [24]. The sequences of gene-specific primers and probes for the *HNMT*, *NSF*, and *PTGS2* genes are presented in Table 1. The specificity of primers and probes was checked using Primer3 and BLAST (https://www.ncbi.nlm.nih.gov/tools/primer-blast/, accessed on 22 August 2023) [36].

Relative gene expression levels in patient samples were calculated using the comparative Ct method *ΔΔ*Ct [37]. The protocol of statistical analysis is described in detail, in the work carried out earlier [33].

## 3. Results

In this study, an analysis of changes in gene expression of *ADORA2A*, *MTA1*, *PTGDS*, *HNMT*, *NSF*, and *PTGS2* at the mRNA level was performed in the peripheral blood from treated and untreated patients with early stages of PD. To assess the specificity of the observed changes for PD pathogenesis, these genes were also analyzed in a neurological control group including patients with various neurodegenerative diseases. The results of the analysis are presented in Table 2.

As is shown in Table 2, significant results in the groups of patients with early stages of PD were obtained for the *ADORA2A*, *MTA1*, *HNMT*, *NSF*, and *PTGS2* genes. Notably, the obtained expression changes are PD-specific, since the expression levels of these genes in the neurological control group do not differ from the expression levels in healthy controls. In the group of untreated patients with early stages of PD, a decrease in the expression level of the *MTA1* gene was detected. An increase of *ADORA2A* gene expression was observed only in a sample of treated patients with early stages of PD. For the *HNMT*, *NSF*, and *PTGS2* genes, there was a significant increase in expression in both the untreated PD patient group and the treated PD patient group. Significant changes in expression of the *PTGDS* gene were found only in the neurological control group.

## 4. Discussion

The study of mRNA levels changes in the blood represents one strategy for finding biomarkers of early stages of PD and may also help to gain insight into the mechanisms of pathogenesis of the disease. In the present study, gene expression changes of *ADORA2A*, *MTA1*, *PTGDS*, *PTGS2*, *NSF*, and *HNMT* were analyzed in the peripheral blood of treated and untreated patients with early stages of PD. In our previous work, we performed a transcriptome analysis in the peripheral blood of three pairs of monozygotic twins discordant for PD, which revealed a significant increase in gene expression of *ADORA2A*, *MTA1*, and *PTGDS* in twins with PD [24]. As shown in Table 2, significant results in the group of patients with early stages of PD were obtained for the *ADORA2A* and *MTA1* genes. The mRNA levels of the *MTA1* and *PTGDS* genes we obtained in the group of patients with early stages of PD did not coincide with the values obtained in the peripheral blood of twins with PD. This result may be explained by the fact that the twins with PD were in more advanced stages of the disease and had received therapy for a longer period than the treated patients at early stages. In contrast, the expression of the *ADORA2A* gene encoding the G-protein-coupled adenosine receptor subtype A2A increased in treated patients with early stages of PD as well as in twins with PD. It is known that adenosine A2A receptors can form heterodimeric complexes with DAergic D2 receptors (DRD2). Furthermore, there are antagonistic interactions between the receptors within these complexes [38]. Since in the present study the increase in *ADORA2A* expression was observed only in treated patients with PD, we assume that this gene is involved in the processes affected by antiparkinsonian drug therapy. This effect may be explained by the fact that the treatment with levodopa or dopamine agonists appears to result in internalization of A2A/DRD2 heterodimers and a compensatory increase in A2A homomers and their signaling. In turn, an increase in *ADORA2A* expression can lead to the development of dyskinesia in treated PD patients [39].

**Figure 1:**
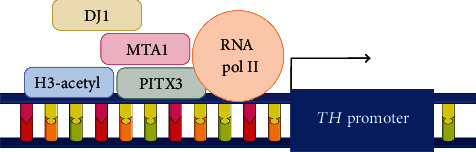
Scheme of the *TH* promoter. MTA1 acts as a coactivator of tyrosine hydroxylase (*TH*) gene transcription through interaction with DJ1, PITX3, Pol II, and chromatin remodeling protein proteins.

The *MTA1* gene encodes metastasis-associated protein 1, which modulates the expression of target genes by functioning as a corepressor or coactivator [40]. In particular, MTA1 functions as a coactivator of the transcription of the tyrosine hydroxylase gene (*TH*), the main enzyme of dopamine synthesis (Figure 1) [41]. Table 2 shows that untreated PD patients showed a statistically significant decrease in *MTA1* transcript levels. At the same time, the expression of *MTA1* increased 1.5-fold in twins with PD relative to healthy twins [24]. The data we obtained can be explained by the fact that the decrease in *MTA1* mRNA levels in the early stages of pathogenesis may be a consequence of DAergic neuronal death. In turn, the increased expression of this gene in twins with later stages of PD is explained by the fact that the cell is trying to compensate for the intensity of dopamine synthesis through increased *TH* expression. Among the published data, only Kumar et al. examined the expression level of *MTA1* in PD, which showed a decrease in *MTA1* expression in the SN in patients with PD [42]. It is important to note that Kumar's work used postmortem brain samples. From this, we can assume that the patients had the late most severe stages of PD, when active medication treatment has been passed and comorbidities may be present. It is likely that at even later stages of the disease, the SN cells no longer have the resources necessary to maintain compensatory mechanisms, which accounts for the decrease in *MTA1* expression.

We also performed expression analysis for the *HNMT*, *NSF*, and *PTGS2* genes selected for analysis based on work previously performed in the laboratory and analysis of published data [25, 26].

**Figure 2:**
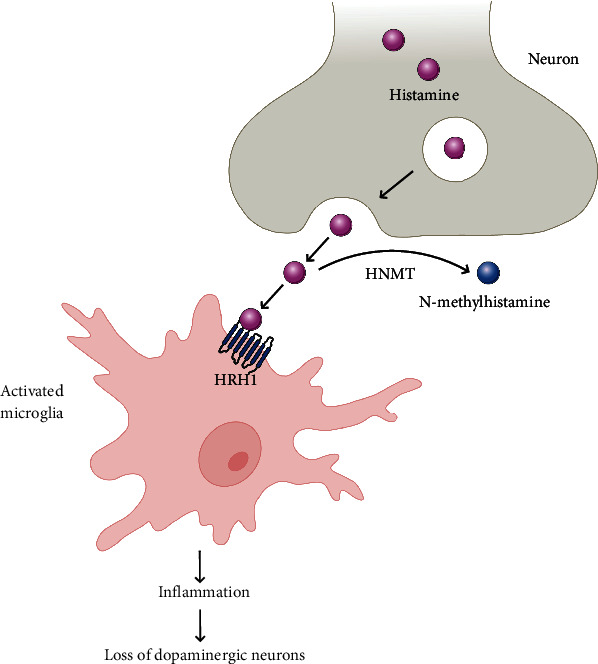
Histamine released from neurons binds to histaminergic receptor H1 (HRH1) on the surface of microglia, thereby leading to its activation. In turn, activation of microglia can lead to the death of DAergic neurons. Some of the released histamine molecules are metabolized to N-methylhistamine by the enzyme histamine-N-methyltransferase (HNMT).

The *HNMT* gene encodes an enzyme of histamine metabolism, histamine N-methyltransferase, which methylates histamine in the presence of S-adenosyl-1-methionine to form N-methylhistamine [43]. In our work, we observed an increase in *HNMT* mRNA levels in the peripheral blood of PD patients. There is evidence that patients with PD are characterized by increased levels of histamine. Thus, increased levels of histamine have been found in postmortem brain samples—in the SN, globus pallidus, and putamen [27]. In addition, elevated histamine levels have been observed in the blood of untreated PD patients [44]. It is known that elevated levels of histamine can contribute to the degeneration of DAergic neurons and trigger inflammatory signaling processes [27, 45]. Histamine through its interaction with histaminergic receptor H1 (HRH1) causes activation of microglia and, eventually, death of DAergic neurons (Figure 2) [46]. In this regard, it can be assumed that the increase in *HNMT* mRNA expression may be a protective mechanism consisting in the enhancement of metabolism of excessive histamine levels. A similar increase in *HNMT* mRNA levels was previously reported in the tissues of SN and putamen in PD patients [47].

In our earlier work focused on the analysis of the expression of transport-associated genes in the brain tissues of mice with the MPTP-induced PD model, interesting results were obtained for the *NSF* gene [26]. We observed a significant increase in mRNA levels of this gene in the SN and striatum tissues in mice with an MPTP-induced model of early PD stage. For this reason, the *NSF* gene became of interest for us to validate the results on human samples. As shown in Table 2, the expression of the *NSF* gene increased significantly in the peripheral blood of patients with early stages of PD. This gene encodes an N-ethylmaleimide-sensitive factor that is involved in intracellular membrane fusion. It performs this function by influencing the assembly and disassembly of the SNARE complex [48]. In particular, *NSF* is involved in neurotransmission, ensuring the fusion of synaptic vesicles to the presynaptic membrane. Evidence is accumulating that *NSF* is involved in PD pathogenesis [49–51]. Our findings on increased *NSF* expression in PD patients may indicate the development of compensatory mechanisms resulting in more active vesicular transport.

The greatest change in expression was obtained for the *PTGS2* gene. The *PTGS2* gene encodes cyclooxygenase-2 (COX2), the main enzyme responsible for the conversion of arachidonic acid into prostaglandin (PG) H2, which is the main precursor of the different PGs, but especially PGE2 [52]. *COX2* expression is known to be mainly upregulated by inflammatory stimuli [53]. At present, there is evidence that *COX2* is involved in the pathophysiology of PD, but its exact role is still unclear [54]. In studies of mice with MPTP-induced models of PD, COX2 has been shown to have deleterious effects on DAergic neurons [55–58]. It is likely that increasing COX2 levels may contribute to neuronal death via dopamine oxidation followed by dopamine-quinone formation or by increasing PGE2 levels, through which the formation of reactive oxygen species and astrocyte activation will occur [52, 59, 60]. Thus, it can be assumed that the increase in *PTGS2* expression that we observed in patients with early stages of PD indicates the development of inflammatory processes, which is likely to lead to even greater death of DAergic neurons and, thereby, enhance neurodegeneration. Our results are consistent with those obtained in some earlier studies. Increased *COX2* expression has been found in the SN of mice with MPTP-induced models of PD, as well as in similar samples from PD patients [56].

**Figure 3:**
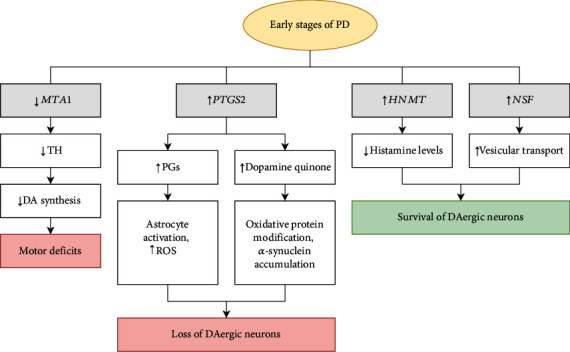
Schematic showing the possible impact of changes in the expression of the genes studied in this paper on the pathogenesis of early stages of PD.

Thus, we found a total of 4 genes that may be associated with early clinical stages of PD. The role of these genes in the pathogenesis of this disease requires further experimental confirmation. Based on the studied literature data and our results, we assume that at the early stages of PD development, *MTA1* and *PTGS2* genes are associated with neurodegenerative processes, while *HNMT* and *NSF* are involved in the development of compensatory effects (Figure 3).

## 5. Conclusions

In summary, analysis of changes in gene expression at the mRNA level in the peripheral blood of patients with early stages of PD showed a significant and PD-specific change in the expression of four genes: *MTA1*, *PTGS2*, *NSF*, and *HNMT*. These genes may be associated with PD pathogenesis in the early clinical stages of the disease. In addition, these genes may be considered as potential biomarkers of PD early stages, but this hypothesis requires further verification in independent patient samples. It is likely that *MTA1* and *PTGS2* are involved in neurodegenerative processes in the early stages of PD pathogenesis, while *HNMT* and *NSF* are involved in the development of compensatory effects. Altered expression of the *ADORA2A* gene in treated PD patients may indicate that this gene is involved in processes affected by antiparkinsonian therapy. The *PTGDS* gene is probably not involved in the pathogenesis of PD in the early stages of pathogenesis.

## Figures and Tables

**Table 1:** Sequences of gene-specific primers and probes.

Gene	Nucleotide sequence
*HNMT (histamine N-methyltransferase)* NM_006895.3^∗^	Probe: 5′-VIC-CAACCATTCCACGGAACACCAGTGC-BHQ2-3′ Forward primer: 5′-ACCACGGGAAATATGTTGAATCTTTC-3′ Reverse primer: 5′-CGCCTATGCTTAGAATCTTAATTTCTGAT-3′

*NSF (N-ethylmaleimide-sensitive factor)* NM_006178.3^∗^	Probe: 5′-VIC-AAGACACATCCATCGGTGGTTCCAGG-BHQ2-3′ Forward primer: 5′-CTCTCCCAATCACAGGTACACATTTA-3′ Reverse primer: 5′-TGCCCAATAGAAAGCCCAGC-3′

*PTGS2 (prostaglandin-endoperoxide synthase 2)* NM_000963.4^∗^	Probe: 5′-VIC-GGTGAAACTCTGGCTAGACAGCGTAAACT-BHQ2-3′ Forward primer: 5′-GCCAGCTTTCACCAACGG-3′ Reverse primer: 5′-TGACTGTGGGAGGATACATCTCT-3′

^∗^Accession numbers in the GenBank database (NCBI-GenBank Release 254.0). VIC: fluorescent dye; BHQ2: fluorescence quencher.

**Table 2:** Results of analysis of changes in relative mRNA levels of studied genes in peripheral blood of patients with early stages of PD (fold change relative to healthy control).

Gene	Untreated patients with PD	Treated patients with PD	Neurological control
*ADORA2A*	1.25^1^ 0.97-1.67^2^	**1.48** **1.10-2.31**	1.25 0.79-1.51

*MTA1*	**0.74** **0.49-1.00**	1.02 0.63-1.71	1.21 1.02-1.49

*PTGDS*	0.67 0.40-1.56	0.89 0.63-1.87	**2.71** **1.95-5.51**

*HNMT*	**1.43** **0.93-2.14**	**1.78** **1.33-1.97**	1.10 0.81-2.04

*NSF*	**1.39** **1.12-1.86**	**1.53** **1.28-2.25**	1.21 0.63-1.78

*PTGS2*	**4.75** **2.70-8.59**	**6.76** **2.51-8.00**	3.20 0.74-8.41

^1^Median. ^2^25-75 percentiles. The data in bold are statistically significant (*p* < 0.05). The expression level in the control is taken as 1.

## Data Availability

The datasets used during the current study are available from the corresponding author on reasonable request.

## References

[1] Balestrino R., Schapira A. H. V. (2020). Parkinson disease. *European Journal of Neurology*.

[2] Lee A., Gilbert R. M. (2016). Epidemiology of Parkinson disease. *Neurologic Clinics*.

[3] Group GBDNDC (2017). Global, regional, and national burden of neurological disorders during 1990-2015: a systematic analysis for the Global Burden of Disease Study 2015. *The Lancet Neurology*.

[4] Dorsey E. R., Constantinescu R., Thompson J. P. (2007). Projected number of people with Parkinson disease in the most populous nations, 2005 through 2030. *Neurology*.

[5] Emamzadeh F. N., Surguchov A. (2018). Parkinson’s disease: biomarkers, treatment, and risk factors. *Frontiers in Neuroscience*.

[6] Poewe W., Seppi K., Tanner C. M. (2017). Parkinson disease. *Nature Reviews. Disease Primers*.

[7] Zeng X. S., Geng W. S., Jia J. J., Chen L., Zhang P. P. (2018). Cellular and molecular basis of neurodegeneration in Parkinson disease. *Frontiers in Aging Neuroscience*.

[8] Kalia L. V., Lang A. E. (2015). Parkinson’s disease. *Lancet*.

[9] Le W., Dong J., Li S., Korczyn A. D. (2017). Can biomarkers help the early diagnosis of Parkinson’s disease?. *Neuroscience Bulletin*.

[10] Amenta F., Bronzetti E., Cantalamessa F. (2001). Identification of dopamine plasma membrane and vesicular transporters in human peripheral blood lymphocytes. *Journal of Neuroimmunology*.

[11] Barbanti P., Fabbrini G., Ricci A. (1999). Increased expression of dopamine receptors on lymphocytes in Parkinson’s disease. *Movement disorders: official journal of the Movement Disorder Society*.

[12] Caronti B., Tanda G., Colosimo C. (1999). Reduced dopamine in peripheral blood lymphocytes in Parkinson’s disease. *Neuroreport*.

[13] Pellicano C., Buttarelli F. R., Circella A. (2007). Dopamine transporter immunoreactivity in peripheral blood lymphocytes discriminates Parkinson’s disease from essential tremor. *Journal of Neural Transmission (Vienna)*.

[14] Buttarelli F. R., Fanciulli A., Pellicano C., Pontieri F. E. (2011). The dopaminergic system in peripheral blood lymphocytes: from physiology to pharmacology and potential applications to neuropsychiatric disorders. *Current Neuropharmacology*.

[15] Blandini F., Sinforiani E., Pacchetti C. (2006). Peripheral proteasome and caspase activity in Parkinson disease and Alzheimer disease. *Neurology*.

[16] Mutez E., Larvor L., Lepretre F. (2011). Transcriptional profile of Parkinson blood mononuclear cells with LRRK2 mutation. *Neurobiology of Aging*.

[17] Karlsson M. K., Sharma P., Aasly J. (2013). Found in transcription: accurate Parkinson’s disease classification in peripheral blood. *Journal of Parkinson's Disease*.

[18] Shamir R., Klein C., Amar D. (2017). Analysis of blood-based gene expression in idiopathic Parkinson disease. *Neurology*.

[19] Shinde S., Pasupathy K. (2006). Respiratory-chain enzyme activities in isolated mitochondria of lymphocytes from patients with Parkinson’s disease: preliminary study. *Neurology India*.

[20] Migliore L., Petrozzi L., Lucetti C. (2002). Oxidative damage and cytogenetic analysis in leukocytes of Parkinson’s disease patients. *Neurology*.

[21] Blandini F., Cosentino M., Mangiagalli A. (2004). Modifications of apoptosis-related protein levels in lymphocytes of patients with Parkinson’s disease. The effect of dopaminergic treatment. *Journal of Neural Transmission*.

[22] Calligaris R., Banica M., Roncaglia P. (2015). Blood transcriptomics of drug-naïve sporadic Parkinson’s disease patients. *BMC Genomics*.

[23] El Haddad S., Serrano A., Moal F. (2020). Disturbed expression of autophagy genes in blood of Parkinson’s disease patients. *Gene*.

[24] Semenova E. I., Vlasov I. N., Partevian S. A. (2022). Transcriptome profiling reveals differential expression of circadian behavior genes in peripheral blood of monozygotic twins discordant for Parkinson’s disease. *Cells*.

[25] Alieva A. K., Rudenok M. M., Novosadova E. V. (2020). Whole-transcriptome analysis of dermal fibroblasts, derived from three pairs of monozygotic twins, discordant for Parkinson’s disease. *Journal of Molecular Neuroscience*.

[26] Rudenok M. M., Shadrina M. I., Filatova E. V. (2022). Expression analysis of genes involved in transport processes in mice with MPTP-induced model of Parkinson’s disease. *Life*.

[27] Anichtchik O. V., Rinne J. O., Kalimo H., Panula P. (2000). An altered histaminergic innervation of the substantia nigra in Parkinson’s disease. *Experimental Neurology*.

[28] Rinne J. O., Anichtchik O. V., Eriksson K. S. (2002). Increased brain histamine levels in Parkinson’s disease but not in multiple system atrophy. *Journal of Neurochemistry*.

[29] Movement Disorder Society Task Force on Rating Scales for Parkinson’s D (2003). The unified Parkinson’s disease rating scale (UPDRS): status and recommendations. *Movement Disorders*.

[30] Goetz C. G., Poewe W., Rascol O. (2004). Movement Disorder Society Task Force report on the Hoehn and Yahr staging scale: status and recommendations. *Movement Disorders*.

[31] Alieva A., Rudenok M., Filatova E. (2020). VCP expression decrease as a biomarker of preclinical and early clinical stages of Parkinson’s disease. *Scientific Reports*.

[32] World Medical A (2013). World medical association declaration of Helsinki. *JAMA*.

[33] Alieva A. K., Filatova E. V., Karabanov A. V., Illarioshkin S. N., Slominsky P. A., Shadrina M. I. (2015). Potential Biomarkers of the Earliest Clinical Stages of Parkinson’s Disease. *Parkinson’s Disease*.

[34] Suslov O., Steindler D. A. (2005). PCR inhibition by reverse transcriptase leads to an overestimation of amplification efficiency. *Nucleic Acids Research*.

[35] Alieva A. K., Filatova E. V., Rudenok M. M., Slominsky P. A., Shadrina M. I. (2021). Housekeeping genes for Parkinson’s disease in humans and mice. *Cell*.

[36] Wheeler D. L., Church D. M., Federhen S. (2003). Database resources of the National Center for Biotechnology. *Nucleic Acids Research*.

[37] Livak K. J., Schmittgen T. D. (2001). Analysis of relative gene expression data using real-time quantitative PCR and the 2(-delta delta C (T)) method. *Methods*.

[38] Schiffmann S. N., Fisone G., Moresco R., Cunha R. A., Ferre S. (2007). Adenosine A_2A_ receptors and basal ganglia physiology. *Progress in Neurobiology*.

[39] Fuxe K., Marcellino D., Genedani S., Agnati L. (2007). Adenosine A(2A) receptors, dopamine D(2) receptors and their interactions in Parkinson’s disease. *Movement Disorders*.

[40] Sen N., Gui B., Kumar R. (2014). Physiological functions of MTA family of proteins. *Cancer Metastasis Reviews*.

[41] Reddy S. D. N., Rayala S. K., Ohshiro K. (2011). Multiple coregulatory control of tyrosine hydroxylase gene transcription. *Proceedings of the National Academy of Sciences of the United States of America*.

[42] Kumar A. S., Jagadeeshan S., Subramanian A. (2016). Molecular mechanism of regulation of MTA1 expression by granulocyte colony-stimulating factor. *The Journal of Biological Chemistry*.

[43] Horton J. R., Sawada K., Nishibori M., Cheng X. (2005). Structural basis for inhibition of histamine N-methyltransferase by diverse drugs. *Journal of Molecular Biology*.

[44] Coelho M. H., Silva I. J., Azevedo M. S., Manso C. F. (1991). Decrease in blood histamine in drug-treated parkinsonian patients. *Molecular and Chemical Neuropathology*.

[45] Liu C. Q., Chen Z., Liu F. X., Hu D. N., Luo J. H. (2007). Involvement of brain endogenous histamine in the degeneration of dopaminergic neurons in 6-hydroxydopamine-lesioned rats. *Neuropharmacology*.

[46] Rocha S. M., Saraiva T., Cristóvão A. C. (2016). Histamine induces microglia activation and dopaminergic neuronal toxicity via H1 receptor activation. *Journal of Neuroinflammation*.

[47] Shan L., Bossers K., Luchetti S. (2012). Alterations in the histaminergic system in the substantia nigra and striatum of Parkinson's patients: a postmortem study. *Neurobiology of Aging*.

[48] Zhao C., Slevin J. T., Whiteheart S. W. (2007). Cellular functions of NSF: not just SNAPs and SNAREs. *FEBS Letters*.

[49] Pischedda F., Cirnaru M. D., Ponzoni L. (2021). LRRK2 G2019S kinase activity triggers neurotoxic NSF aggregation. *Brain*.

[50] Belluzzi E., Gonnelli A., Cirnaru M. D. (2016). LRRK2 phosphorylates pre-synaptic N-ethylmaleimide sensitive fusion (NSF) protein enhancing its ATPase activity and SNARE complex disassembling rate. *Molecular Neurodegeneration*.

[51] Babcock D. T., Shen W., Ganetzky B. (2015). A neuroprotective function of NSF1 sustains autophagy and lysosomal trafficking in Drosophila. *Genetics*.

[52] Teismann P. (2012). COX-2 in the neurodegenerative process of Parkinson’s disease. *BioFactors*.

[53] Rumzhum N. N., Ammit A. J. (2016). Cyclooxygenase 2: its regulation, role and impact in airway inflammation. *Clinical and Experimental Allergy*.

[54] Minghetti L. (2004). Cyclooxygenase-2 (COX-2) in inflammatory and degenerative brain diseases. *Journal of Neuropathology and Experimental Neurology*.

[55] Feng Z. H., Wang T. G., Li D. D. (2002). Cyclooxygenase-2-deficient mice are resistant to 1-methyl-4-phenyl 1, 2, 3, 6-tetrahydropyridine-induced damage of dopaminergic neurons in the substantia nigra.

[56] Teismann P., Tieu K., Choi D. K. (2003). Cyclooxygenase-2 is instrumental in Parkinson’s disease neurodegeneration. *Proceedings of the National Academy of Sciences of the United States of America*.

[57] Teismann P., Ferger B. (2001). Inhibition of the cyclooxygenase isoenzymes COX-1 and COX-2 provide neuroprotection in the MPTP-mouse model of Parkinson's disease. *Synapse*.

[58] Aubin N., Curet O., Deffois A., Carter C. (1998). Aspirin and salicylate protect against MPTP-induced dopamine depletion in mice. *Journal of Neurochemistry*.

[59] Hastings T. G. (1995). Enzymatic oxidation of dopamine: the role of prostaglandin H synthase. *Journal of Neurochemistry*.

[60] Chae S. W., Kang B. Y., Hwang O., Choi H. J. (2008). Cyclooxygenase-2 is involved in oxidative damage and alpha-synuclein accumulation in dopaminergic cells. *Neuroscience Letters*.

